# Different socialities affect habitat preferences in the coexisting wood-feeding cockroaches *Panesthia angustipennis spadica* and *Salganea esakii*

**DOI:** 10.1038/s41598-025-09257-8

**Published:** 2025-07-03

**Authors:** Hiroki Ito, Naoya Osawa

**Affiliations:** 1 Laboratory of Forest Ecology, Graduate School of Agriculture, Kyoto University, Kyoto, 606-8502 Japan; 2 Laboratory of Applied Entomology, Faculty of Agriculture, Setsunan University, Osaka, Japan

**Keywords:** Coarse Woody debris, Colony composition, Gregariousness, Ovoviviparous insect, Xylophagy, Ecology, Behavioural ecology, Forest ecology

## Abstract

Wood-feeding cockroaches generally have long nymphal periods and adult lifespans. Especially for social species, stable and durable habitats may be required for taking care of their offspring in the same place. We compared the preferences of habitats, colony composition, and reproduction in two coexisting wood-feeding cockroaches, the gregarious *P. angustipennis spadica* and the subsocial *S. esakii*, in a temperate forest. Colonies of *P. angustipennis spadica* were more frequently found in wood at higher decay stages, while the number of individuals of *S. esakii* was larger in wood at lower decay stages. In *P. angustipennis spadica*, nymphs in all size classes were found without adults, suggesting that dispersed nymphs prefer more highly decayed wood, which is easy to digest and nutrient-rich. On the other hand, *S. esakii* form larger colonies with multiple broods, indicating that they may prefer less-decayed wood for maintaining colonies over several years. Thus, the different intensities of sociality in wood-feeding insects may play a role in the decomposition process of decayed woods in forests.

**Supplementary Information:**

The online version contains supplementary material available at 10.1038/s41598-025-09257-8.

## Introduction

In social insects, breeding location is important for maintaining kin groups and caring for offspring, and especially for making stable nests from spatially fragmented resources^1^. In the plant-ant *Pseudomyrmex concolor* Smith, colony size is primarily limited by the domatium space offered by the host plant^2^. Moreover, ants in decayed wood form smaller colonies than those living in less restricted sites, such as the open soil, ground surface, and tree canopy^3^. Making a new nest is costly, as moving dropped eggs or offspring to a new nest may be difficult for many species, although nest movement has been reported in ants, termites, bees, and wasps^4^. In addition to eusocial (highest level of sociality with castes and division of labor, colonies with adults of two generations, and cooperative activity^5^) termites, subsocial (caring for their own immature offspring over a period of time^5^) wood-feeding species with biparental care are known in Blattodea and Coleoptera^6^. In wood-feeding insects, a wood diet may select for parental care in three ways: (1) nymphs receive symbiotic microorganisms, for digesting wood, from their parents; (2) wood is physically difficult to process for younger nymphs; and (3) the low nitrogen availability in wood causes slow nymphal growth^7^. Furthermore, Maekawa et al.^8^ described that living in a food source (i.e., decayed wood) allows parents of wood-feeding cockroaches to forage without abandoning their nymphs, and the interiors of logs are safe and easy to defend from predators. Therefore, social wood-feeding insects in wood must select resources with enough space to maintain colonies, suitable materials for digging tunnels, and moderate environmental conditions, such as temperature and moisture, over the period of parental care.

*Panesthia angustipennis spadica* Shiraki and *Salganea esakii* Roth (Blattodea: Blaberidae) are wood-feeding cockroaches in the subfamily Panesthiinae^9^. Both species coexist sympatrically in Kyushu, Japan and its surrounding islands, and they bore tunnels and reside in decayed wood^10^. In all areas where *S. esakii* has been recorded, *P. angustipennis spadica* has also been recorded, but *P. angustipennis spadica* is the only wood-feeding cockroach in Honshu and Shikoku Islands, Japan^10^. *P. angustipennis spadica* is macropterous, and adults may have the ability to fly, while brachypterous *S. esakii* adults cannot fly^10^. Wood-feeding Panesthiinae produces their own cellulases^11^and may not require symbiotic microorganisms for cellulose decomposition^12^. Parental feeding has not been observed in genus *Panesthia*. Adult pairs with nymphs have been found in *Panesthia cribrata* Saussure and *P. angustipennis spadica*, but colonies with plural male and/or female adults were also found^13,14^. In addition, *P. angustipennis spadica* colonies composed of small nymphs without adults are often found in fields^15,16^. Interestingly, first-instar nymphs without mothers grew faster than those with mothers^17^. These studies showed that the intensity of socialities in *P. angustipennis spadica* varies widely and that parental care is not always necessary for nymphs, suggesting that *P. angustipennis spadica* is gregarious but not subsocial^18^. In *Salganea taiwanensis* Roth and *S. esakii*, stomodeal trophallaxis between parents and nymphs has been observed^19,20^. Moreover, 39.3–85.0% of colonies in *Salganea* spp. contained adult and nymph family individuals, and 22.7–55.0% were biparental families^8^suggesting that *Salganea* spp., including *S. esakii*, are subsocial. Wood-feeding cockroaches are relatively large in body size among the wood-feeding insects^21^and they progressively degrade the logs which they inhabit^22^. Therefore, habitat preferences in wood-feeding cockroaches may affect decomposition processes in forest ecosystems.

In subsocial wood-feeding insects, the restriction of using wood as a nutrition source may cause a parental care requirement, and nesting in decayed woods enables adults to maintain colonies and protect offspring. Therefore, the establishment of subsociality in wood-feeding cockroaches may be related to their food and nest resources. However, the size and decay stages of wood vary widely in a natural forest, and social and non-social species may prefer different characteristics of decaying woods. In this study, we investigated colony composition and reproduction schedule in relation to the preferences of decayed wood for two coexisting wood-feeding cockroaches, *P. angustipennis spadica* and *S. esakii*, to clarify the relationship between socialities and habitat preferences.

## Materials and methods

### Study site

Field surveys were conducted in part of a chinquapin oak forest in Fukuregi National Forest (32º 24’ N, 130º 05’ E) in Shimoshima, Amakusa Island, Kumamoto, Japan. This secondary forest was mainly covered by trees (under ca. 30 cm in chest-height diameter), including *Quercus gilva* Blume, *Quercus salicina* Blume, *Quercus sessilifolia* Blume, *Distylium racemosum* Siebold & Zuccarini, and *Meliosma rigida* Siebold & Zucc.

### Preferences of decayed woods

We prepared seven semicircle plots (radius of 15 m, slope-area ca. 353.25 m^2^) on a west-facing slope (ca. 200 m × 100 m) in Fukuregi National Forest, and data were collected for a total of six times, in 2014 (July and September), 2015 (May, July, and September), and 2016 (May). In these plots, we collected the wood-feeding cockroaches *P. angustipennis spadica* and *S. esakii*, ants, and termites from all accessible decayed woods (with max. diameter over 5 cm, *n* = 375), including from standing decayed wood, stumps, and fallen logs. The small nymphs of *P. angustipennis spadica* and *S. esakii* can be easily distinguished in the field because the former have dark and opaque cuticle, but the latter have pale and transparent cuticle, respectively^18^. We collected wood-feeding cockroaches for each colony, with colony members defined as individuals sharing the same gallery (*P. angustipennis spadica*: *n* = 39; *S. esakii*: *n* = 32). To collect all individuals of wood-feeding cockroaches in each decayed log, we laid a plastic sheet under logs, and broke it into pieces (for soft parts) or peeled the bark and traced tunnels (for hard parts), using knives and trowels. We recorded the presence of ants and termites only when more than five individuals were found in each decayed log. We measured diameter by tape measure (to the nearest 1 mm, for both ends and middle of the largest trunk or branch) and decay class (from 1 to 5) of all decayed woods^23^. For disintegrated logs, diameter values were recorded as semicircular cross-sections (max. mean diameter: 25.3 cm; min: 4.0 cm). The decay classes were determined by knife penetration length (at the middle of decayed wood by a 5 mm thick blade) and by appearance of the decayed woods (Table 1). In addition, colonies of wood-feeding cockroaches were collected outside the plots, and the decay classes of the woods where they were found were recorded in the same way (*P. angustipennis spadica*: *n* = 4; *S. esakii*: *n* = 90).

To clarify the relationship between colony composition and decayed-wood preference, we distinguished the stage (adult or nymph) and sex (only for adults) of collected individuals and classified colonies of each species into five categories: adults (only adult(s) except adult pair), adult pairs, adults and nymphs, nymphs, and solitary nymphs.

### Colony composition

In addition to collecting samples for studying wood preferences, we collected colonies of wood-feeding cockroaches (*P. angustipennis spadica*: *n* = 22; *S. esakii*: *n* = 49) outside the plots in 2015 (May, July, and September) and 2016 (January and May) without recording the woody characteristics or presence of other insects. We measured the pronotum widths of collected wood-feeding cockroaches (*P. angustipennis spadica*: *n* = 112; *S. esakii*: *n* = 733) using a digital caliper (ca. > 10 mm) and a micrometer with stereoscopic microscope (ca. < 10 mm). Some collected individuals had broken pronotums (due to failed molts or injuries caused by field collection and those not), so we could not measure their pronotum widths (*P. angustipennis spadica*: *n* = 3; *S. esakii*: *n* = 29) and only recorded their stages (adult or nymph) and sexes (for adults).

Data for mean pronotum width for each instar of laboratory-reared nymphs were used to estimate the instars of field-collected nymphs. Data for *P. angustipennis spadica* was drawn from Ito and Osawa^17^ using nymphs born from field-collected parents in Kyoto, Japan, and data for *S. esakii* was drawn from Obata^15^ using nymphs born from field-collected parents and field-collected nymphs (for “final”) in Yakushima Island, Japan.

To estimate the number of broods in each colony of *S. esakii*, we calculated the max. / min. ratio of nymphal pronotum width in each colony. We considered a colony to have multiple broods when the max. / min. ratio of nymphal pronotum width was higher than 1.7, because Obata^15^ reported mean pronotum width in each instar (first to seventh instar in laboratory rearing) and found the ratios between adjacent instars to be 1.23 to 1.30. When the largest nymph was 1.7 (> 1.69 = 1.30 squared) times larger than the smallest nymph, then instars of both individuals were expected not to be next to each other.

### Reproduction

**Fig. 1:**
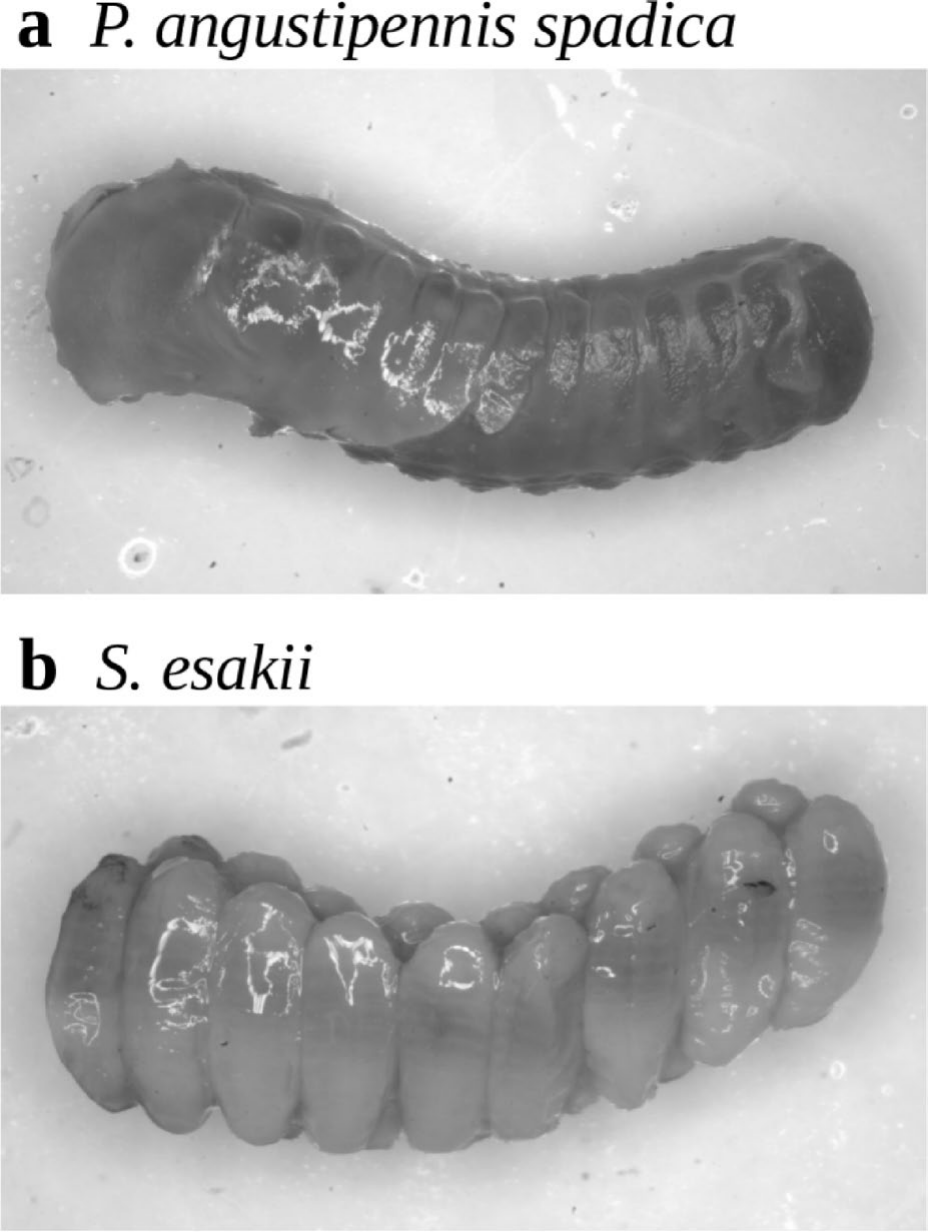
Oothecae of **a** *P. angustipennis spadica* and **b** *S. esakii* in complete shape. Eggs are often scattered in early stages.

We dissected collected female adults of *P. angustipennis spadica* and *S. esakii* and counted the number of eggs in their brood sacs. To estimate reproductive season, we stored some female adults in ethanol immediately after field collection and dissected them in the laboratory (*P. angustipennis spadica*: *n* = 6; *S. esakii*: *n* = 48) (Fig. 1). Mature eggs were found in female adults collected in May and July, but ootheca shapes were often scattered in immature stage in May (*P. angustipennis spadica*: 100%, *n* = 4; *S. esakii*: 66.7%, *n* = 8). We kept other female adults of *S. esakii* (collected in May 2016) in plastic containers (182 × 112 × 107 mm) with wood pieces in the laboratory; when we killed them in July 2016, we found completely-shaped ootheca in seven female adults. We then counted the number of eggs from only female adults died in July (*P. angustipennis spadica*: *n* = 1; *S. esakii*: *n* = 22). Newly emerged or aged adults were inferred by checking the wing shape in *P. angustipennis spadica*, because this species has long wings (ca. 30 mm length) at the newly emerged stage, but its wings are often reduced by the aged stage by rubbing while burrowing in woody tunnels.

**Table 1:** Decay classes. Adapted from Heilmann–Clausen (2001)

Decay class	Knife penetration length	Appearance	
		Bark	Circumference shape
1	Less than 3 mm	Intact	Intact
2	3 mm–1 cm	Starting to break up	Intact
3	1–5 cm	Partly lost	Intact
4	5–10 cm	Lost in most places	Disintegrated
5	More than 10 cm*	Lost in most places	Disintegrated

*For logs with a diameter of 10 cm or less were classified as decay class five when the knife penetrated completely.

**Table 2:** Result of hurdle model for the number of individuals of each wood-feeding cockroach species in each decayed wood.

	Estimate	SE	Z value	*P* value
*P. angustipennis spadica*				
Count model coefficients (truncated poisson with log link)				
Intercept	0.138	0.314	0.439	0.661
Diameter	0.264	0.135	1.959	0.050
Decay class [4 and 5]*	− 0.176	0.359	− 0.490	0.624
Ant	− 1.018	0.428	− 2.377	0.017
Termite	1.312	0.331	3.969	< 0.001
* S. esakii*	− 0.885	0.444	− 1.993	0.046
Zero hurdle model coefficients (binomial with logit link)				
Intercept	− 3.101	0.328	− 9.469	< 0.001
Diameter	0.833	0.162	5.129	< 0.001
Decay class [4 and 5]*	0.878	0.406	2.165	0.030
Ant	0.510	0.412	1.237	0.216
Termite	0.149	0.549	0.272	0.786
* S. esakii*	1.213	0.482	2.513	0.012
*S. esakii*				
Count model coefficients (truncated poisson with log link)				
Intercept	1.367	0.159	8.589	< 0.001
Diameter	0.201	0.123	1.632	0.103
Decay class [4 and 5]*	− 0.675	0.341	− 1.981	0.048
Ant	− 0.265	0.232	− 1.140	0.254
Termite	− 1.216	0.362	− 3.359	< 0.001
* P. angustipennis spadica*	0.294	0.237	1.242	0.214
Zero hurdle model coefficients (binomial with logit link)				
Intercept	− 2.771	0.291	− 9.524	< 0.001
Diameter	0.548	0.169	3.247	0.001
Decay class [4 and 5]*	− 0.613	0.501	− 1.223	0.221
Ant	0.326	0.455	0.717	0.474
Termite	0.506	0.524	0.967	0.334
* P. angustipennis spadica*	1.122	0.487	2.304	0.021

*Decay class was divided two categories: 1−3 vs. 4 and 5.

**Table 3:** Colony composition in the field.

Categories of colony	% (*n*)	
	*P. angustipennis spadica*	*S. esakii*
Colonies with adults	9.2 (6)	73.9 (133)
An adult pair and nymphs	1.5 (1)	21.7 (39)
A male adult and nymphs	1.5 (1)	3.9 (7)
A female adult and nymphs	3.1 (2)	10.6 (19)
An adult pair	1.5 (1)	28.9 (52)
Only Adults	1.5 (1)	8.9 (16)
Colonies without adults	90.8 (59)	26.1 (47)
Nymphs	30.8 (20)	6.1 (11)
Solitary nymph	60.0 (39)	20.0 (36)

### Statistical analysis

For decayed wood preferences, we used the hurdle model to analyze the number of individuals of each wood-feeding cockroach species in each decayed wood as a function of the mean diameter, the decay class of decayed wood (decay class: two categories 1−3 vs. 4 and 5), and the presence of ants, termites, and other species of wood-feeding cockroach using the *hurdle* function in the *R* package *pscl* v1.5.5 ^24^. The hurdle model is a two-component model, including a count model and a zero-hurdle model, and which is suitable for analyzing data with excess of zeroes in the objective variable (the number of individuals of each cockroach species were zero in 89.6% decayed woods for *P. angustipennis spadica*, and in 91.5% for *S. esakii*). In this study, the impacts of explanatory variables on number of individuals were tested by the count model, and their impacts on the presence of cockroaches as a binary variable were tested by the zero-hurdle model^24,25^. A Fisher’s exact test was used to compare the inhabitant rates of each wood-feeding cockroach species between woods of different decay classes in the plots. A chi-squared test was also used to compare the inhabitant rates at each decay class between *P. angustipennis spadica* and *S. esakii* in the plots. Differences in decay class between colony categories were compared using a Kruskal–Wallis test. A Wilcoxon rank-sum test was used to compare colony sizes (number of individuals in a colony) between *P. angustipennis spadica* and *S. esakii*. We also performed a two-sample test for equality of proportions with continuity correction to compare adult ratio.

All the analyses were conducted using *R* v3.6.3 ^26^, and the significance level *P* was set at 0.05.

## Results

### Preferences of decayed woods

**Fig. 2:**
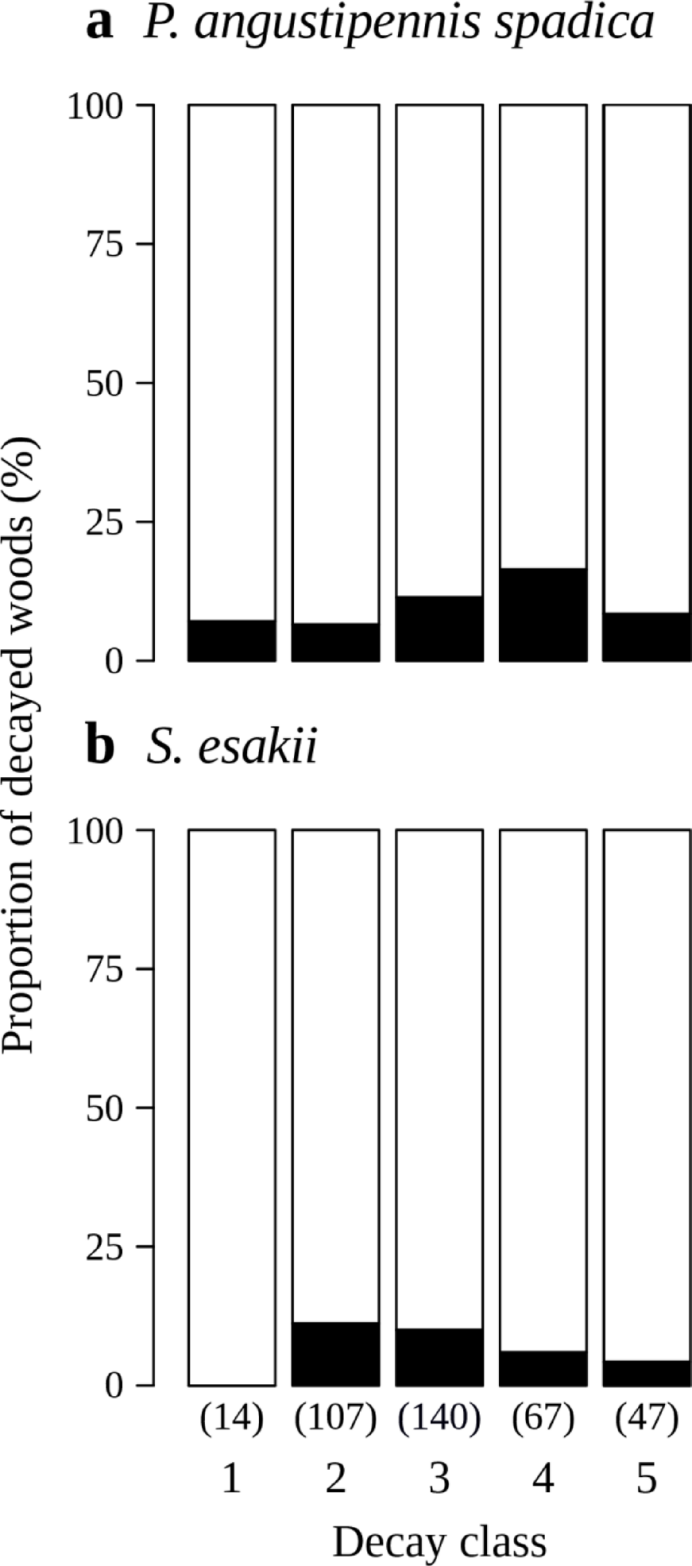
Occurrence of **a*** P. angustipennis spadica* and **b** *S. esakii* found in decaying woods of different decay classes in the plots. Black and white boxes refer to proportions of decaying woods with and without wood-feeding cockroaches, respectively. Numbers of decayed woods observed are given in parentheses.

*P. angustipennis spadica* and *S. esakii* were collected in 10.4% and 8.5% of the decayed woods in the plots, respectively, and both species coexisted in 2.9% of the woods. In total, wood-feeding cockroaches were living in 16.0% of the decayed woods, and this rate was higher than for termites (10.4%). Moreover, wood-feeding cockroaches were collected from various decay classes (*P. angustipennis spadica*: decay classes 1−5; *S. esakii*: 2−5) (Fig. 2). Adult *S. esakii* pairs without nymphs were found only from decay classes two and three in the plots. *P. angustipennis spadica* was collected from decayed woods with 12.69 ± 0.76 (mean ± standard error [SE]) cm mean diameter (*n* = 39), and *S. esakii* was collected from 12.25 ± 0.82 cm mean diameter logs (*n* = 32).

**Fig. 3:**
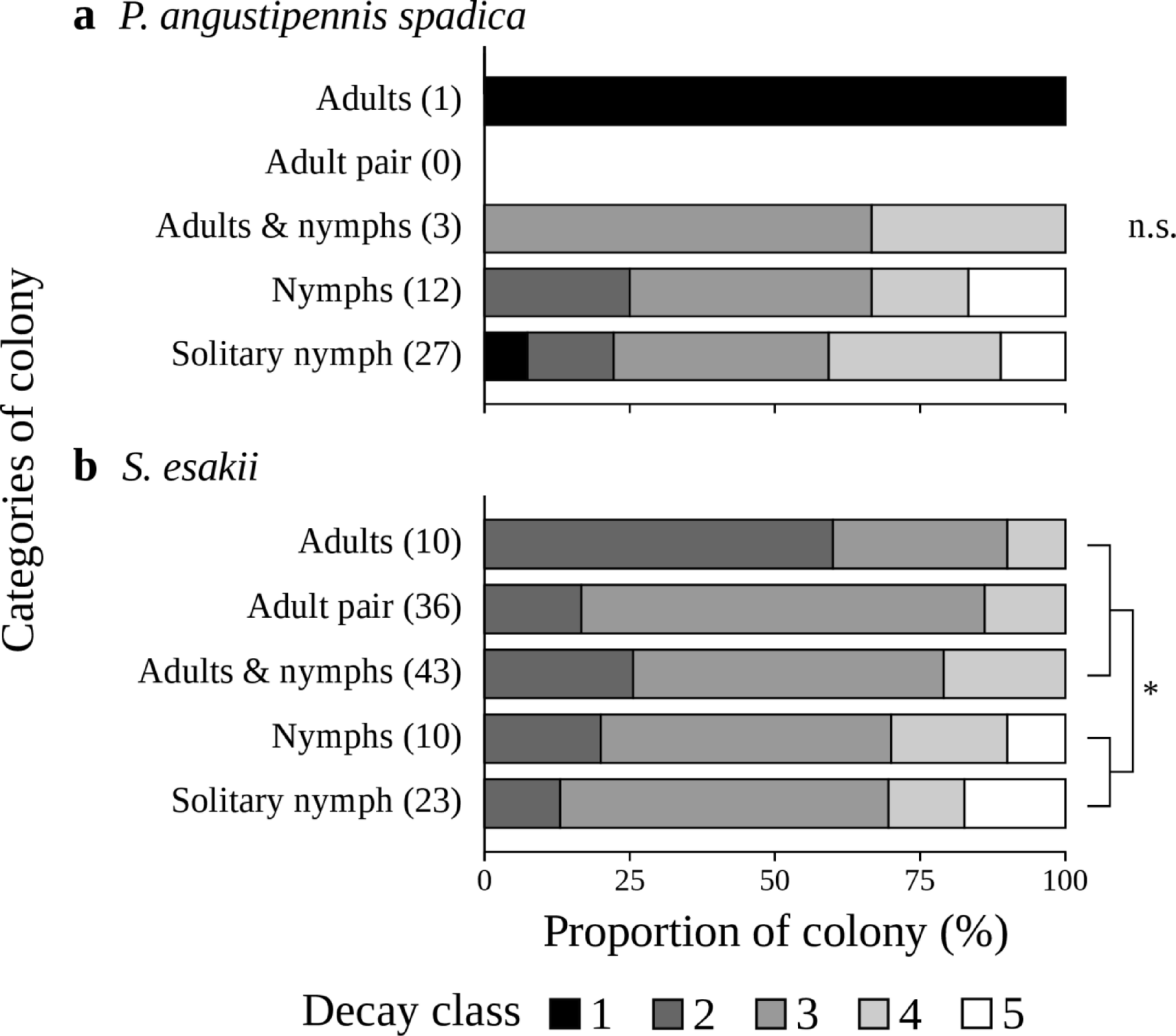
Proportions of **a** *P. angustipennis spadica* and **b** *S. esakii* colonies found in wood of different decay classes. Numbers of colonies in each category are given in parentheses. *P < 0.05; n.s. non-significance in all combinations of categories (Kruskal–Wallis test) diameter.

The zero-hurdle model results indicate a significantly increased likelihood of observing (*P* < 0.001 and *P* = 0.001) for decayed woods with larger mean diameters for *P. angustipennis spadica* and *S. esakii*, respectively (Table 2). The zero-hurdle model results also indicate that a higher decay class and the presence of *S. esakii* significantly (*P* = 0.030 and *P* = 0.012, respectively) increased the probability of *P. angustipennis spadica* occurrence and that the presence of *P. angustipennis spadica* significantly (*P* = 0.021) increased the *S. esakii* inhabitant rate, but that decay class did not influence *S. esakii* (*P* = 0.221) (Table 2). For *P. angustipennis spadica*, the count model results show that the number of individuals was significantly affected by several factors: the presence of termites had a positive effect (*P* < 0.001), and the presence of ants and *S. esakii* (*P* = 0.017 and *P* = 0.046, respectively) had negative effects (Table 2). On the other hand, the count model results for *S. esakii* indicate that the presence of termites and a higher decay class (*P* < 0.001 and *P* = 0.048, respectively) had negative effects on the number of individuals (Table 2). When focusing on only decay class, the inhabitant rates of wood-feeding cockroaches in the plots were not significantly different in either species (decay class 1−3 vs. 4 and 5) (Fisher’s exact test, *P* = 0.271 in *P. angustipennis spadica*; *P* = 0.162 in *S. esakii*) (Fig. 2), but the inhabitant rates of adult pairs of *S. esakii* without nymphs were significantly higher in lower decay classes (Fisher’s exact test, *P* = 0.021). The frequencies of cockroach-inhabited decayed woods at different decay classes (decay class 1−3 vs. 4 and 5) were marginally significantly different (chi-squared test, χ^2^ = 3.279, d.f. = 1, *P* = 0.070), and the rates of cockroach-inhabited decayed woods in lower decay classes (1−3) tended to be higher for *S. esakii* than for *P. angustipennis spadica*. There was no significant difference for *P. angustipennis spadica* (Kruskal–Wallis test, *χ*^2^ = 0.430, d.f. = 1, *P* = 0.512), while decay classes of decaying wood utilized by colonies with adults were significantly lower than those utilized by colonies containing only nymphs in *S. esakii* (Kruskal–Wallis test, *χ*^2^ = 4.094, d.f. = 1, *P* = 0.043) (Fig. 3).

### Colony composition

**Fig. 4:**
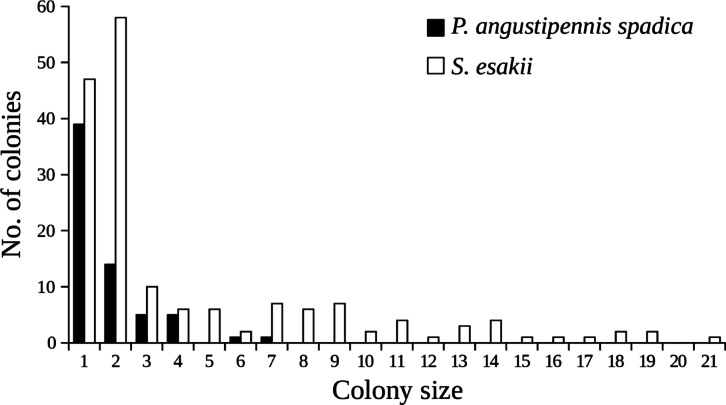
Colony sizes in *P. angustipennis spadica* and *S. esakii*.

Colony size was smaller for *P. angustipennis spadica* than for *S. esakii* (*P. angustipennis spadica*: 1.77 ± 0.16 [mean ± SE], *n* = 65; *S. esakii*: 4.29 ± 0.35, *n* = 171; Wilcoxon rank-sum test, W = 7734, *P* < 0.001) (Fig. 4). The adult ratio of *S. esakii* was also higher than for *P. angustipennis spadica* (*P. angustipennis spadica*: 8.7%; *S. esakii*: 23.4%; two-sample test for equality of proportions with continuity correction, *χ*^2^ = 20.842, d.f. = 1, *P* < 0.001).

**Fig. 5:**
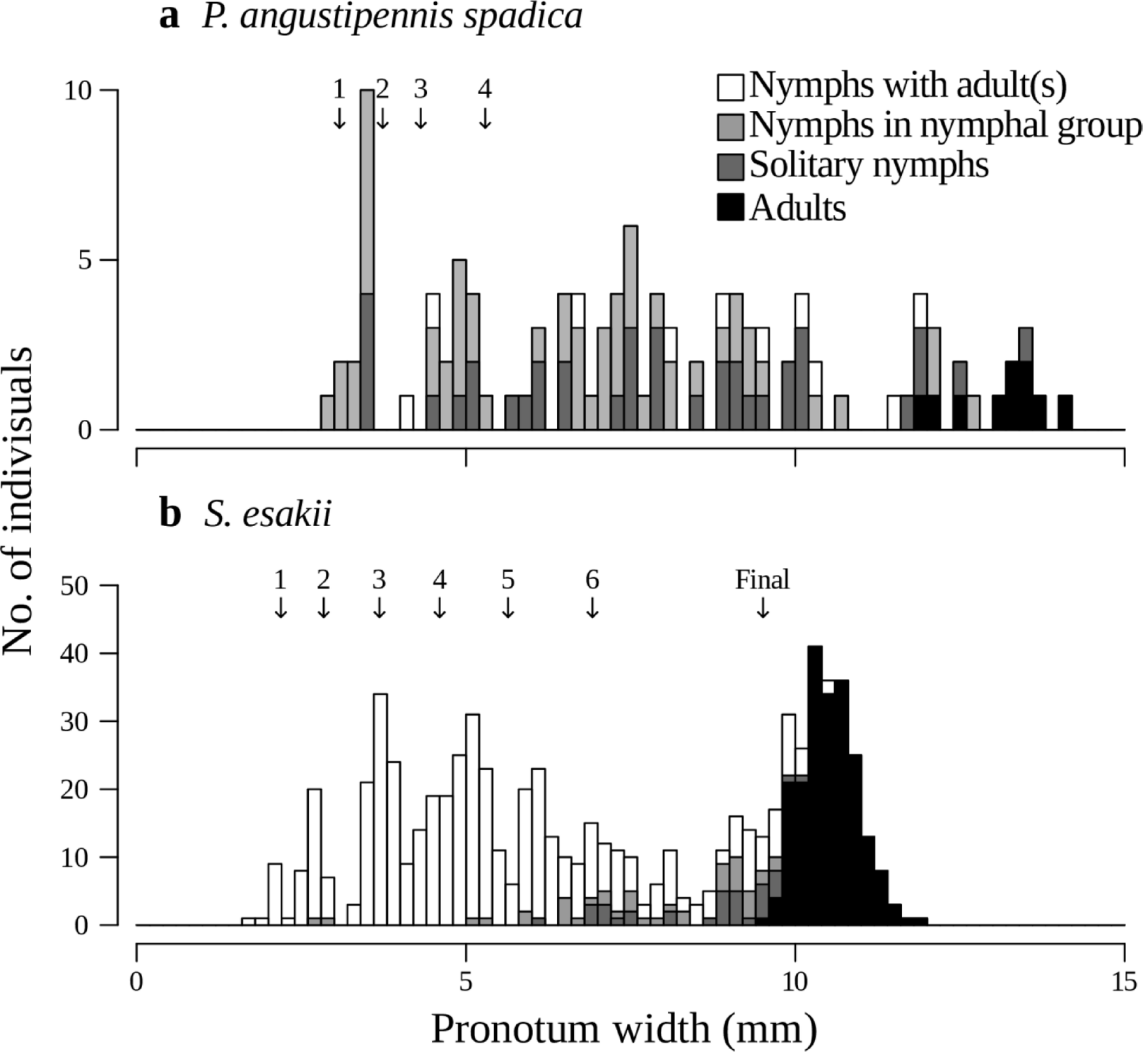
Frequency distribution of pronotum width (at 0.2 mm intervals) in **a** *P. angustipennis spadica* and **b** *S. esakii*. Arrows and numbers (and “Final”) indicate mean pronotum width in each instar in laboratory rearing as determined by previous study (see Materials and methods).

**Fig. 6:**
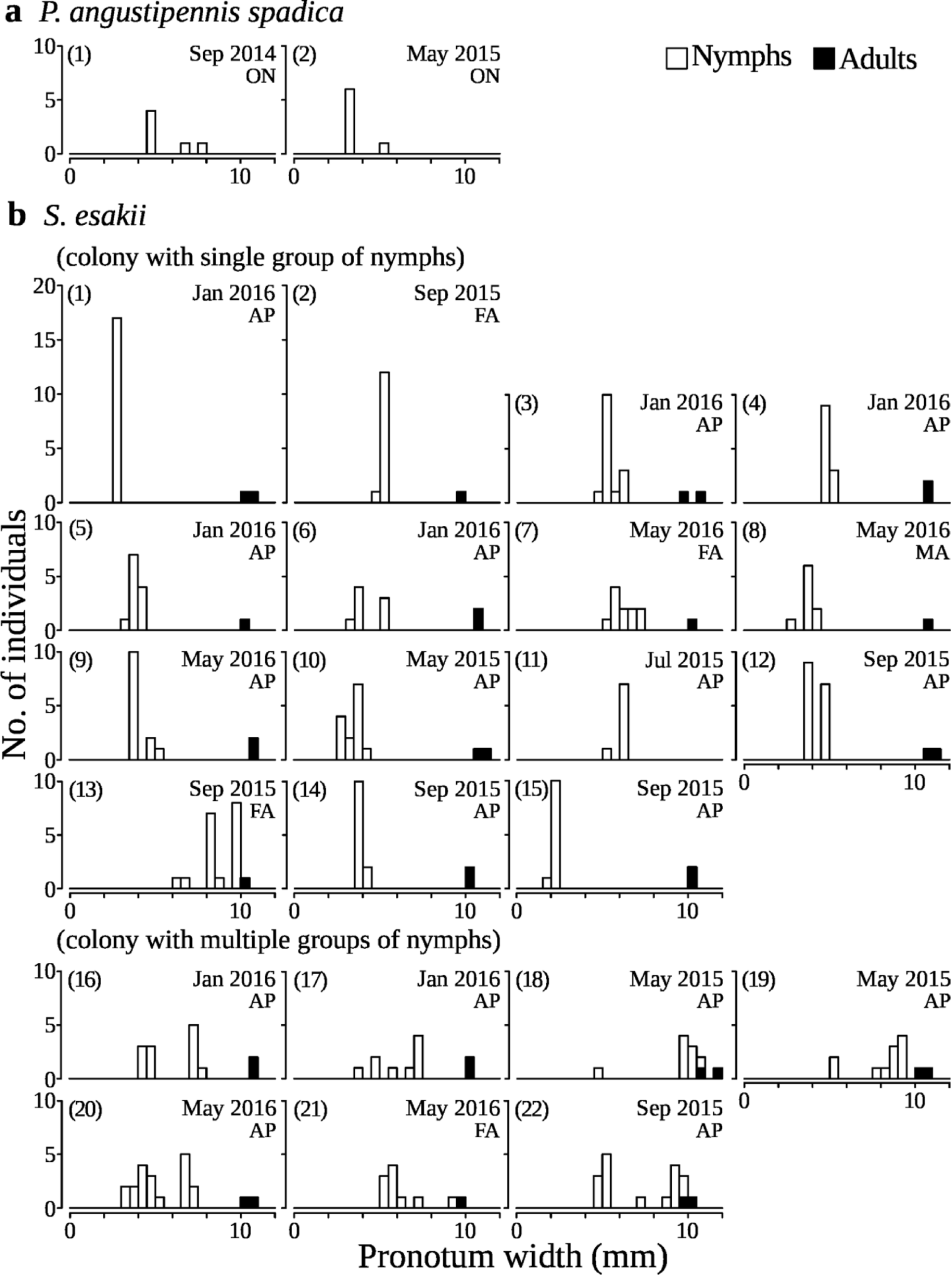
Size distribution in each colony of **a** *P. angustipennis spadica* (colony size ≥ 5) and **b** *S. esakii* (colony size ≥ 10). Different numerals indicate different colonies: Nos. 1 and 2 in **a** and Nos. 1−22 in **b**. The collection date is shown in the upper right, and categories of colony (AP: An adult pair and nymphs; MA: A male adult and nymphs; FA: A female adult and nymphs; ON: Only nymphs) are shown under the collection date. In **b** Nos. 4 and 15, pronotum width was not measured for one individual in each colony, and two were not measured in **b** No. 11. Each histogram cell contains individuals separated by 0.5 mm in pronotum width. In **b** *S. esakii*, Nos. 1−15 contained single group of nymphs of similar sizes (max / min ratio in nymphal pronotum width < 1.7), and Nos. 16−22 contained multiple groups of nymphs.

In *P. angustipennis spadica*, 90.8% of colonies were without adults (Table 3), and the body sizes of nymphs without adults varied widely from the smallest to the largest classes of collected nymphs (Fig. 5). On the contrary, 26.1% colonies of *S. esakii* were without adults (Table 3), and young nymphs (< 6 mm in pronotum width, estimated less than five instar) without adults were rare (2.0%, *n* = 6) (Fig. 5). Moreover, adult pairs (with or without nymphs) were found in 50.6% (an adult pair and nymphs: 21.7% plus an adult pair without nymphs: 28.9%) of *S. esakii* colonies (Table 3). The largest colony of *P. angustipennis spadica* contained only nymphs, while all large colonies (≥ 10 individuals, *n* = 22) of *S. esakii* were families, with 15 colonies containing a single group of nymphs of similar body sizes and seven colonies containing multiple broods of nymphs (Fig. 6).

In eleven colonies, *P. angustipennis spadica* and *S. esakii* shared the galleries: two contained an adult and nymphs of *P. angustipennis spadica* and one *S. esakii* nymph, three colonies contained a family (an adult pair and nymphs) of *S. esakii* and a few nymphs (one to three individuals) of *P. angustipennis spadica*, three contained an adult pair of *S. esakii* and a few nymphs (one or two individuals) of *P. angustipennis spadica*, and three contained only nymphs of both species.

### Reproduction

**Fig. 7:**
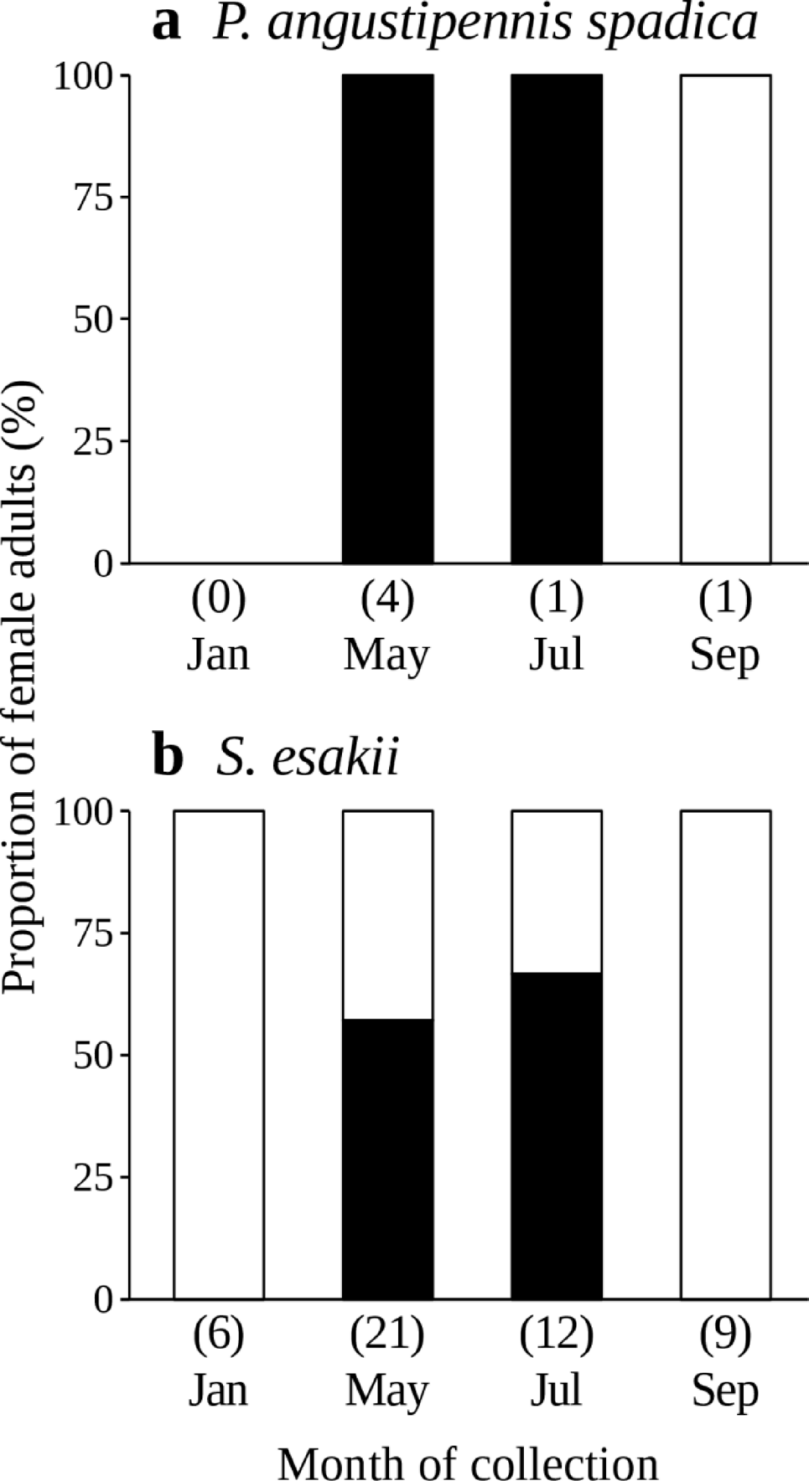
Proportions of female adults with eggs in each month in **a** *P. angustipennis spadica* and **b** *S. esakii*. Black and white boxes refer to rates of female adults with and without eggs, respectively. Numbers of individuals are given in parentheses.

In both *P. angustipennis spadica* and *S. esakii*, female adults with eggs were collected only in May and July (Fig. 7). All collected female adults of *P. angustipennis spadica* had eggs in May and July (Fig. 7), and female adults of *P. angustipennis spadica* with full wings (inferred to be newly emerged adults) were included among them (*n* = 2). However, 39.4% of *S. esakii* female adults collected in May and July did not have eggs (*n* = 13) (Fig. 7), and adult pairs without nymphs were found in all collected months (*n* = 6 in January, *n* = 38 in May, *n* = 7 in July, *n* = 1 in September). Female adults of *S. esakii* with nymphs also had eggs (*n* = 9).

**Fig. 8:**
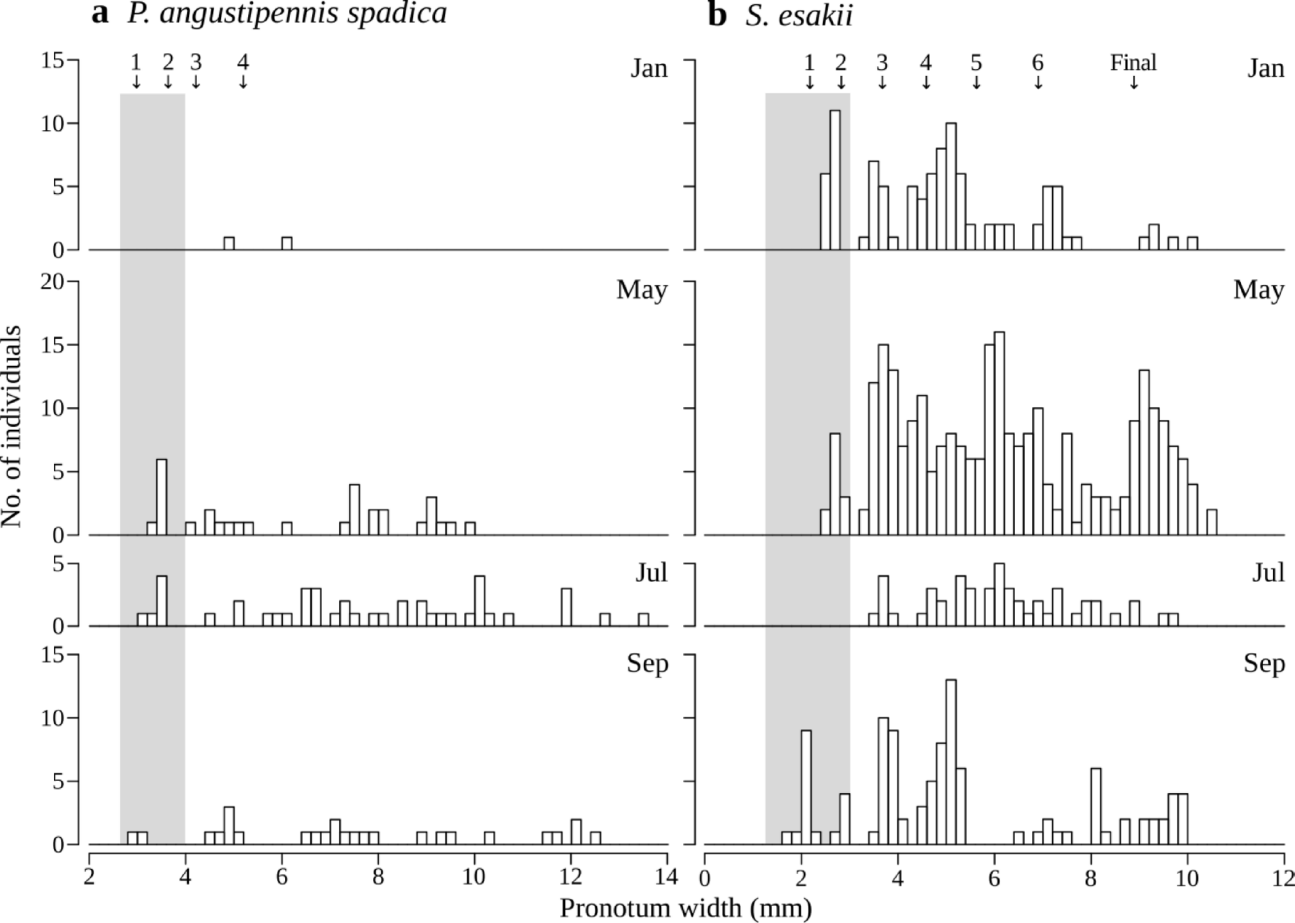
Frequency distribution of pronotum width (at 0.2 mm intervals) in **a** *P. angustipennis spadica* and **b** *S. esakii* nymphs in each month. The collection month is shown in the upper right. Arrows and numbers (and “Final”) indicate mean pronotum width in each instar in laboratory rearing as determined by previous study (see Materials and Methods). Shaded areas indicate small nymphs for **a** *P. angustipennis spadica*: ≤ 4 mm in pronotum width; and **b** *S. esakii*: ≤ 3 mm.

In *P. angustipennis spadica*, small nymphs (≤ 4 mm in pronotum width) were collected in May, July, and September, and the smallest nymphs were collected in September (Fig. 8). The small nymphs (≤ 3 mm in pronotum width) of *S. esakii* were collected in January, May, and September, and the nymphs in the smallest group (estimated as first instar) were found in September (Fig. 8). On the other hand, large nymphs (estimated as final instar) of *S. esakii* were found in all collected months (Fig. 8).

The number of eggs in an ootheca was 23 in *P. angustipennis spadica* (*n* = 1) and 14.00 ± 0.85 (mean ± SE) in *S. esakii* (*n* = 14).

## Discussion

### The role of *P. angustipennis spadica* and *S. esakii* in the wood decomposition process

In this plots, wood-feeding cockroaches were living in 16.0% of decayed woods, and they were collected from all decay classes (Fig. 2), indicating that *P. angustipennis spadica* and *S. esakii* utilize decayed woods of a wide range of decay classes. These findings suggest that cockroaches may play a certain role in the decomposition of coarse woody debris in a forest. Wood-feeding insects may also introduce microorganisms into the interiors of logs when they dig tunnels^27^. In addition, insects living inside logs may lead to fragmentation of decayed woods by larger predators searching for prey^28^.

### Preferences of decayed woods related to the intensity of sociality

This study showed that inhabitant rates of both *P. angustipennis spadica* and *S. esakii* increased in decayed woods with larger diameters (Table 2). In both species, nymphal and adult periods last for several years^8,29^. Frangi et al. (1997)^30^ showed a negative correlation between decay rate and diameters of branches and boles in *Nothofagus pumilio* Poepp. & Endl. Moreover, micro-climatic conditions may be more stable in woods of larger diameter because of their lower surface-to-volume ratio and thicker bark^30^. Therefore, decayed woods with larger diameters may be preferable for both *P. angustipennis spadica* and *S. esakii*, which live long-term inside decayed wood. On the other hand, Edmonds & Eglitis (1989)^31^ reported that large-diameter logs of *Pseudotsuga menziesii* (Mirb.) Franco decomposed faster than smaller ones due to the presence of the wood-boring beetle *Monochamus scutellatus* Say, which was only observed in large-diameter logs. Their diameter preferences may accelerate the decomposition of larger decayed woods and influence the nutrient cycle in the forest, although quantitative information about decomposition by wood-feeding cockroaches is unknown.

The hurdle model result showed that the probability of *P. angustipennis spadica* occurrence increased in higher decay classes (Table 2). The inhabitant rate of *P. angustipennis spadica* tended to be the highest in decay class four (Fig. 2). Moreover, mean and maximum colony sizes of *P. angustipennis spadica* were smaller than for *S. esakii* (Fig. 4), and nymphs of *P. angustipennis spadica* in all size classes were found without adults (Fig. 5). In *P. angustipennis spadica*, no parental care has been observed^19^and small nymphs without adults in the fields have been reported in previous studies^14,18^. In addition, first-instar nymphs of *P. angustipennis spadica* have rather well-developed cuticles and eyes^18^and they may have higher wood-digesting abilities^11^ than does *S. esakii*. Furthermore, nymphs of *P. angustipennis spadica* grow faster in solitary conditions^17^. These findings indicate that developmental and physiological characteristics may enable young nymphs of *P. angustipennis spadica* to be independent from their parents. Typically, the concentration of nutrients (e.g., N, P, and Mg) in decaying wood increases during the decomposition process^32^and the materials become softer during decaying and easier for small nymphs to crush with their chin. Therefore, *P. angustipennis spadica* may prefer logs of higher-decaying wood, which are nutrient-rich and easy to digest.

The number of *S. esakii* individuals was lower for higher decay classes (Table 2), and the decay class with the highest inhabitant rate of *S. esakii* was class two in the plots (Fig. 2). Adult pairs without nymphs were found only in decay classes two and three in the plots. Moreover, the decay classes of decayed woods with adults were significantly lower than for colonies without adults in *S. esakii* (Fig. 3). Klass et al. (2008)^33^ observed that the subsocial wood-feeding cockroaches *Cryptocercus* spp. nest in harder and more durable decayed woods, while *Parasphaeria boleiriana* Grandcolas & Pellens, with less social behaviors, feeds on soft and ephemeral wood sources. In *S. esakii*, it is expected that host-decayed woods for families are chosen by parents and that productive colonies will last several years in the same log. In this study, at least seven colonies of *S. esakii* seemed to contain multiple broods (Fig. 6), and we found that nine female adults of *S. esakii* with nymphs had eggs. Families of *Salganea* spp. with multiple broods have also been reported in previous works^15,34^and most studied *Salganea* spp. appear to reproduce once per year^8^. Therefore, *S. esakii* adult pairs may prefer decayed woods in lower decay classes when beginning colonization because harder logs remain for longer periods. In this case, large colonies tended to be found in lower decay classes, so the number of individuals was high in lower decay classes. Colonies of *S. esakii* were also found in decay classes four and five (Fig. 2), and colonies without adults utilized decayed woods in higher decay classes than did colonies with adults (Fig. 3). In addition, in *S. esakii* most nymphs without adults were larger (≥ 6 mm in pronotum width) individuals (Fig. 5), and no nymphal group containing many individuals (≥ 10) was found in this study. These results suggest that larger nymphs may sometimes disperse from their parents before emergence and select highly decayed woods as temporary habitats. Hövemeyer & Schauermann (2003)^35^ reported that branches (4.3–11.5 cm in diameter) of *Fagus sylvatica* L. in a temperate forest remained at less than 20% dry weight (estimated as decay class 5 in our study) ten years after the trees had died. Therefore, another possibility is that nymphs without adults have been abandoned by parents or their parents have died, and the decay stage of host decayed woods have progressed during their nymphal period.

The presence of another wood-feeding cockroach had a positive effect on inhabitant rate for both *P. angustipennis spadica* and *S. esakii* (Table 2). In the plots, either or both *P. angustipennis spadica* and *S. esakii* were collected from 16.0% of decayed woods, and both species were living in 18.1% of them (2.9% of total), indicating that the habitats of the two species are overlapping, and their preferences for host-decayed woods may be similar in terms other than diameter. However, the number of individuals of *P. angustipennis spadica* decreased in the decayed woods with *S. esakii*, while the presence of *P. angustipennis spadica* did not significantly affect the number of *S. esakii* in the same decayed woods (Table 2). In the subsocial wood-feeding cockroach *Cryptocercus punctulatus* Scudder, adults and large nymphs frequently fight against members of different families^36^. We found eleven galleries shared by *P. angustipennis spadica* and *S. esakii*, but adults of both species did not coexist in the same gallery. Although there is no precise information about the behavior of *P. angustipennis spadica* and *S. esakii* inside decayed woods, it is possible that adults of *P. angustipennis spadica* and *S. esakii* avoid reproducing near the nests of other species. In this case, the small number of dispersed nymphs of *P. angustipennis spadica* arrive at decayed woods nested by *S. esakii*, resulting in a decreasing number of individuals of *P. angustipennis spadica* in decayed woods with *S. esakii* (Table 2). In two galleries, solitary nymphs of *S. esakii* were found with an adult and nymphs of *P. angustipennis spadica*, implying that dispersed nymphs of *S. esakii* can also arrive at decayed woods nested by *P. angustipennis spadica*, but the rate of dispersion from their parents may be much lower than for *P. angustipennis spadica* (Table 3; Fig. 5). This may cause no significant difference in the number of individuals of *S. esakii* between decayed woods with and without *P. angustipennis spadica* (Table 2).

The number of individuals of *P. angustipennis spadica* increased in decayed woods with termites but decreased in woods with ants (Table 2). In addition, the presence of termites had a negative effect on the number of *S. esakii* individuals (Table 2). Both termites and ants may compete with wood-feeding cockroaches for nesting resources, and ants can also be predators of wood-feeding cockroaches. It is known that termites and wood-nesting ants also have preferences for decayed woods^37,38^and the wood-nesting ant *Crematogaster ashmeadi* Mayr utilizes cavities bored by other wood-boring insects such as moth larvae and termites^39^. We do not know how wood-feeding cockroaches are affected by the presence of termites and ants, when choosing host decayed woods, and this area requires further research.

### Factors of variations at colony composition of *P. angustipennis spadica* and the role of *S. esakii*

In this study, we collected 65 colonies of *P. angustipennis spadica*. The maximum colony size was seven (Fig. 4), and only 6.1% colonies contained adults and nymphs (Table 3). However, a previous study in Kyoto, Japan, where *S. esakii* does not live, showed that four out of 33 colonies contained more than 20 individuals, with a maximum colony size of 65, and that 39.4% colonies contained adults and nymphs^14^. These differences between two studied sites may be caused by differences in the distribution patterns of decayed woods or in the density of *P. angustipennis spadica*^22^. The hurdle model results showed that the presence of *S. esakii* had a negative effect on the number of individuals of *P. angustipennis spadica* in each decayed wood (Table 2). Therefore, the presence of *S. esakii* may also play a role in modifying the colony composition of *P. angustipennis spadica* in a field population. In this study, the habitats of *P. angustipennis spadica* and *S. esakii* overlapped, so it is possible that interspecific competition causes a lower density of *P. angustipennis spadica*. In addition, six nymphal colonies of *P. angustipennis spadica* were observed in galleries with adults of *S. esakii* in this study. Wood-feeding cockroaches have relatively large body sizes among insects living in decayed woods^21^and their galleries are not closed. Moreover, many young nymphs of *P. angustipennis spadica* were smaller in pronotum width than adults of *S. esakii* (Fig. 5). This suggests that adults of *S. esakii* might be gallery providers for dispersed *P. angustipennis spadica* nymphs. The woody tunnels burrowed by adults of *S. esakii* might increase the survival rates of dispersed *P. angustipennis spadica* nymphs, because the woody tunnels may be safer against predators than staying on the surface of logs. Thus, the presence of *S. esakii* might cause smaller colony sizes and higher rates of colonies without adults in *P. angustipennis spadica*.

### Reproduction schedule in *P. angustipennis spadica* and *S. esakii*

Female adults with eggs were only found in May and July (Fig. 7), and the smallest nymphs were found in September for *P. angustipennis spadica* and *S. esakii* (Fig. 8). Therefore, both species may reproduce mainly in July to September in this study site. Park et al. (2002)^40^ reported that the nymphs of *Cryptocercus kyebangensis* Grandcolas stop growing during winter in a temperate mountain forest. In laboratory rearing, molting of *P. angustipennis spadica* was not observed in the winter season (Ito unpublished data). Therefore, small nymphs collected in January and May might have been born in the previous summer to autumn (Fig. 8).

The number of eggs in an ootheca was higher for *P. angustipennis spadica* than for *S. esakii*. Similar clutch sizes were observed in another location^15^. Gregarious *P. angustipennis spadica* does not perform parental care. In laboratory, *P. angustipennis spadica* was reported to reproduce twice in a year^17^. Furthermore, female adults estimated to have newly emerged had eggs in this study. This circumstantial evidences suggest that female adults of *P. angustipennis spadica* might be able to start reproduction earlier than *S. esakii*. In addition to their larger body size, their lesser sociality may enable *P. angustipennis spadica* to produce more nymphs in a reproductive season. On the other hand, the costs of parental care may limit the number of offspring in *S. esakii*^41^. Among the *S. esakii* colonies, 28.9% were adult pairs without nymphs (Table 3), and adult pairs without nymphs were found in all collected months. Furthermore, 39.4% of female adults of *S. esakii* did not have eggs in May or July (Fig. 7). Adult pairs of *C. punctulatus* form in late spring to early autumn and reproduce the next summer in a temperate mountain forest^42^. These findings suggest that adult pairs of *S. esakii* spend a long time together before first reproduction, just like *C. punctulatus*, in order to prepare nest galleries or wait until a favorable season for reproduction, implying that adult pairs of *S. esakii* should choose durable nest sites. Otherwise, adult pairs without eggs or nymphs may have lost all their offspring for accidental reasons (e.g., predation, disease, and/or destruction of nest galleries).

## Electronic supplementary material

Below is the link to the electronic supplementary material.

Supplementary Material 1.

Supplementary Material 2.

Supplementary Material 3.

## Data Availability

Data is provided within the manuscript and Supplementary materials files.
