# Supplementary material for: Different socialities affect habitat preferences in the coexisting wood-feeding cockroaches Panesthia angustipennis spadica and Salganea esakii
Source: Sci Rep. 2025 Jul 3;15:23722. doi: 10.1038/s41598-025-09257-8 (PMC12229330; doi:10.1038/s41598-025-09257-8)
Supplement: Supplementary file 1 — Supplementary Material 1. [file 41598_2025_9257_MOESM1_ESM.docx]

cockroaches.csv

The data of field collected individuals of wood-feeding cockroaches in Amakusa Island.

[Item name]

[Descriptions]

[Legend]

[Example]

Year-Month

date of field collection

[Year]-[Month]

2014-07

Label

Individuals with same label are member of one colony

[Colony ID]

A-1-1

Species

Species

"*S. esakii*" / "*P. angustipennis spadica*"

*S. esakii*

Sex

Sex of individuals (not recorded for small nymphs)

"Male" / "Female" / "-"

Male

Stage

Developmental stage

"Adult" / "Nymph"

Adult

PronotumWidth

Pronotum width (mm) (not recorded for some individuals)

[Pronotum width (mm)] / "-"

2.16

WingLength

Wing length (recorded for only adults of *P. angustipennis spadica*)

[Wing length (mm)] / "-"

28.39

WingStatus

Wing status (recorded for only adults of *P. angustipennis spadic*a

"Full" / "Reduced" / "-"

Full

Egg

Number of eggs (recorded for some female adults)

[Number of eggs] / "-"

14

EggStatus

Egg status (recorded for only female adults with eggs)

"Mature" / "Immature" / "-"

----------

log.csv

The data of decayed logs in Amakusa Island.

[Item name]

[Descriptions]

[Legend]

[Example]

DecayClass

Decay class (from 1 to 5) of the decayed log (class 1 is hardest and 5 is softest).

"1" / "2" / "3" / "4" / "5"

"1"

Diameter

Mean diameter (cm).

[Mean diameter (cm)]

7.2

Ant

Presence or not of ants.

"0": Not presence / "1": Presence

1

Termite

Presence or not of termites.

"0": Not presence / "1": Presence

1

Esakii

Presence or not of *S. esakii*.

"0": Not presence / "1": Presence

0

Spadica

Presence or not of *P. angustipennis spadica*.

"0": Not presence / "1": Presence

0

EsakiiAdultPair

Presence or not of *S. esakii* adult pairs without nymphs.

"0": Not presence / "1": Presence

0
